# A Novel Topology of Proline-rich Transmembrane Protein 2 (PRRT2): HINTS FOR AN INTRACELLULAR FUNCTION AT THE SYNAPSE*

**DOI:** 10.1074/jbc.M115.683888

**Published:** 2016-01-21

**Authors:** Pia Rossi, Bruno Sterlini, Enrico Castroflorio, Antonella Marte, Franco Onofri, Flavia Valtorta, Luca Maragliano, Anna Corradi, Fabio Benfenati

**Affiliations:** From the ‡ Department of Experimental Medicine, University of Genova, Viale Benedetto XV, 3, 16132 Genova, Italy,; the § Center for Synaptic Neuroscience and Technology, Fondazione Istituto Italiano di Tecnologia, Largo Rosanna Benzi 10, 16132 Genoa, Italy, and; the ¶ San Raffaele Scientific Institute, Vita Salute University, Via Olgettina 58, 20132 Milan, Italy

**Keywords:** membrane protein, molecular dynamics, neurological disease, SNARE proteins, synapse

## Abstract

Proline-rich transmembrane protein 2 (PRRT2) has been identified as the single causative gene for a group of paroxysmal syndromes of infancy, including epilepsy, paroxysmal movement disorders, and migraine. On the basis of topology predictions, PRRT2 has been assigned to the recently characterized family of Dispanins, whose members share the two-transmembrane domain topology with a large N terminus and short C terminus oriented toward the outside of the cell. Because PRRT2 plays a role at the synapse, it is important to confirm the exact orientation of its N and C termini with respect to the plasma membrane to get clues regarding its possible function. Using a combination of different experimental approaches, including live immunolabeling, immunogold electron microscopy, surface biotinylation and computational modeling, we demonstrate a novel topology for this protein. PRRT2 is a type II transmembrane protein in which only the second hydrophobic segment spans the plasma membrane, whereas the first one is associated with the internal surface of the membrane and forms a helix-loop-helix structure without crossing it. Most importantly, the large proline-rich N-terminal domain is not exposed to the extracellular space but is localized intracellularly, and only the short C terminus is extracellular (N_cyt_/C_exo_ topology). Accordingly, we show that PRRT2 interacts with the Src homology 3 domain-bearing protein Intersectin 1, an intracellular protein involved in synaptic vesicle cycling. These findings will contribute to the clarification of the role of PRRT2 at the synapse and the understanding of pathogenic mechanisms on the basis of PRRT2-related neurological disorders.

## Introduction

Proline-rich transmembrane protein 2 (PRRT2) is encoded by a gene that has been shown recently to be the single causative gene for a group of paroxysmal syndromes of infancy, including benign familial infantile seizures, infantile convulsion choreoathetosis, migraine, hemiplegic migraine, and paroxysmal kinesigenic dyskinesia/choreoathetosis. A large number of PRRT2 nonsense, frameshift, and missense mutations have been associated with diseases with a variable phenotypic spectrum, ranging from mild forms that improve with age to severe phenotypes (1–5).

The PRRT2 gene encodes for a protein highly conserved across species, with an average of 80% similarity across mammals and 30% with zebrafish. The sequence similarity increases in the C-terminal region containing the predicted transmembrane domains, becoming over 90% in mammals and 60% in zebrafish (6). On the basis of topology prediction, PRRT2 has been assigned to the newly characterized large family of Dispanins, whose members share the two-transmembrane domain topology and include the subfamily of interferon-induced transmembrane proteins.

The putative topology of the Dispanins has been described as two transmembrane helices in the C-terminal region, separated by an intracellular loop of variable length, and an often long N-terminal region (>100 amino acids) compared with a short C-terminal region (<10 amino acids) that are both oriented toward the outside of the cell (7). A detailed analysis of the primary structure of human PRRT2 identified the two predicted transmembrane helices in the C-terminal region of the protein (TM1,^3^ amino acids 275–295; TM2, amino acids 324–344): a long and relatively unstructured N-terminal region (amino acids 1–274), including a proline-rich domain (amino acids 133–222); an intracellular loop (amino acids 296–323) connecting the two transmembrane helices; and a very short C-terminal end (8). The mouse PRRT2 ortholog is virtually identical to the human protein in the C terminus, with few differences in the N-terminal region (6).

Many PRRT2 mutations are highly penetrant and cause truncation of the protein. The most frequent mutation is a recurrent frameshift located in an instable region of nine cytosines, causing the introduction of a stop codon seven amino acids downstream of insertion (c.649–650insC > p.Arg217Profs*7). A variety of other nonsense or frameshift mutations are mostly located in the long N-terminal domain of the protein, scattered in the proline-rich domain, and only a few involve the TM domains or the cytoplasmic loop. The truncated mutations are degraded by nonsense-mediated mRNA decay or by protein degradation, as suggested by the lack of their expression in cell lines (9–16).

PRRT2 mRNA is expressed in the nervous system in the cortex, hippocampus, basal ganglia, and cerebellum (6, 7, 9), which are all regions putatively involved in the pathogenesis of PRRT2-linked diseases. The function of PRRT2 is characterized poorly. However, a link with the machinery of neurotransmitter release has been hypothesized on the basis of results of proteomic and biochemical studies showing an interaction with synaptosome-associated protein 25 kDa (SNAP-25) (6, 17, 18), one of the presynaptic SNARE proteins that constitute the fusion core complex allowing exocytosis of synaptic vesicles (19). In addition, a high-resolution proteomics study revealed the presence of PRRT2 among new auxiliary proteins participating in the AMPA glutamate receptor complex, with a preferential association of PRRT2 with the AMPA receptor subunit GLUA1 (20). This putative interaction has been confirmed recently (18). In addition, the observed colocalization of exogenously expressed PRRT2 with synaptic markers further suggests its targeting to synaptic areas (6). All of this experimental evidence points to a functional role of PRRT2 at the synapse.

If the topology model of the Dispanin protein family, with both the N- and C-terminal domains exposed to the external surface of the cell, also holds true for PRRT2, it may implicate that the long PRRT2 N-terminal domain interacts with proteins of the extracellular matrix and/or with extracellular domains of synaptic proteins and works as a synaptic adhesion molecule. Several trans-synaptic interacting proteins, such as the neurexin-neuroligin or the cadherin-catenin complexes, integrins, integrin ligands, and LGI1, play crucial roles in synapse formation and functioning and are causative genes of synaptopathies such as epilepsy and/or autism (21–24).

On the contrary, if the orientation of the N terminus with respect to the plasma membrane were different, then a completely diverse set of putative protein interactors and other functions could be hypothesized. In this work, we found that PRRT2 has a different topology with respect to the one predicted for the other Dispanins. Through a series of complementary experimental approaches such as live immunolabeling, immunogold electron microscopy, surface biotinylation, and computational modeling, we demonstrate that the large N-terminal domain is not exposed to the extracellular space but that it is cytosolic and that only the very short C terminus is extracellular (N_cyt_/C_exo_ orientation). This topology conforms to that of type II transmembrane proteins with a very short C-terminal anchor and leads to the conclusion that only the second C-terminal hydrophobic stretch spans the plasma membrane, whereas the first one is associated with the inner surface of the membrane without crossing it.

## Experimental Procedures

### Cell Culture Procedures

COS7 cells were cultured in advanced DMEM supplemented with 10% fetal bovine serum, 1% l-glutamine, 100 units/ml penicillin, and 100 μg/ml streptomycin (Life Technologies) and maintained at 37 °C in a 5% CO_2_ humidified atmosphere. Cells were transfected with Lipofectamine 2000 (Life Technologies).

### Constructs

We generated three distinct HA constructs using the pKH3 plasmid (a gift from Ian Macara, Addgene plasmid 12555) (25): HA_3_ tags at the N terminus of PRRT2 (HA-PRRT2), with the PRRT2 mouse coding sequence cloned in BamH1-EcoR1 sites; HA_3_ tags at the C terminus of PRRT2 (PRRT2-HA), with the PRRT2 mouse coding sequence without a stop codon cloned in Hind3-Sal1; and HA_3_ tags between the two hydrophobic transmembrane segments in the putative cytosolic loop of the protein (PRRT2-loop-HA), with PRRT2 1–894 and PRRT2 895–1041 cloned in Hind3-Sal1 and BamH1-EcoR1 sites, respectively. For biotinylation experiments, PRRT2-HA was used as template to obtain a mutant (PRRT2ΔC) lacking the C-terminal tail (PRRT2 with a stop codon after Val-344) by site-directed mutagenesis (forward primer 5′-CAT CAA CTT AGG CGT GTA GAA GGT CGA CTC TAG AA-3′) using the QuikChange Lightning site-directed mutagenesis kit (Agilent). To generate PRRT2 lacking the N-terminal domain (PRRT2ΔN), the sequence from 799 to 1039 of the mouse cDNA was cloned by PCR in Hind3-Sal1 sites of the pkH3 plasmid with the following primers: forward, AGATAAGCTTATG GGCACCCAGAAACCTCGGGAC; reverse, CACTGTCGACCTTATACACGCCTAAGTTGATGACGCA. In addition, Lys-270 was replaced with an arginine by site-directed mutagenesis (forward primer 5′-ATGGGCACCCAGAGACCTCGGGACTATATC). This construct retains the HA_3_ at the C terminus of PRRT2. For the GFP construct, the PRRT2 mouse coding sequence was cloned in the pEGFP-C2 plasmid (BD Biosciences). Mouse PRRT2 fused at the C terminus with turboGFP was purchased from Origene. All primers were purchased from Eurofins Genomics (Ebersberg, Germany).

### Immunocytochemistry

Primary hippocampal neurons were fixed with 4% paraformaldehyde in PBS (pH 7.4) and 4% sucrose. Cells were permeabilized with 0.1% Triton X-100 in PBS for 5 min and blocked with 0.1% Triton X-100 and 4% fetal bovine serum in PBS for 30 min. Samples were incubated sequentially with primary antibodies in blocking solution (3 h at room temperature), followed by Alexa Fluor 488- or 594-conjugated secondary antibodies (Invitrogen, 1:500 for 1 h at room temperature) (26). Coverslips were mounted using Prolong Gold antifade reagent (Invitrogen) and DAPI for nuclear staining. Images were captured with an Olympus BX41 epifluorescence microscope (×20/0.5 and ×40X/0.75 objectives, Olympus) equipped with a QIClick-F-M-12 charge-coupled device camera (QImaging).

### Live Immunolabeling

Cells transfected with the various PRRT2 constructs were live-labeled by diluting primary antibodies (rabbit anti-PRRT2, 1:200, Sigma-Aldrich; mouse anti-HA, 1:200, Invitrogen; mouse anti-turboGFP, 1:200, Origene; and mouse anti-GFP, 1:500, Millipore) in culture medium for 1 h at 37 °C in a 5% CO_2_ incubator to detect the surface epitopes, followed by incubation with Alexa Fluor 488 or 594 secondary antibodies for 1 h at 37 °C, and fixed. In the experiments of live-labeling with HA antibodies, after live incubation with Alexa Fluor 488 secondary antibodies, cells were fixed with 4% paraformaldehyde, permeabilized with 0.1% Triton X-100, and stained with anti-PRRT2 antibodies, followed by Alexa Fluor 594 secondary antibodies to label total PRRT2.

### Western Blotting

These experiments were performed as described previously (27). Primary antibodies were as follows: rabbit anti-PRRT2 (1:500, Sigma-Aldrich), mouse anti-actin (1:1000, Sigma-Aldrich), mouse anti-Na^+^/K^+^ATPase (1:1000, Millipore), and mouse anti-dynamin I (1:1000, Millipore).

### Surface Biotinylation Assays

COS7 cells were transfected with PRRT2-HA-, PRRT2ΔC-, or PRRT2ΔN-expressing plasmids. The biotinylation assay was performed as described previously (28).

### Electron Microscopy

#### 

##### Pre-embedding Immunogold Technique

COS7 cells were transiently transfected with DNA encoding either HA-PRRT2 or PRRT2-HA. Pre-embedding immunogold labeling was performed 48 h after transfection. Cells were fixed in 4% paraformaldehyde in 0.1 m PBS (pH 7.4), permeabilized and incubated with rabbit anti-HA (1:100, Bethyl Laboratories). Samples were then incubated with a secondary antibody (goat anti-rabbit, 1:100, Nanoprobes) conjugated to a colloidal nanogold particle (10 nm). Cells were then fixed for 1 h in 1.2% glutaraldehyde in PBS, post-fixed in 1% osmium tetroxide in 0.1 m sodium cacodylate (pH 7.4), *en block* stained in 0.5% uranyl acetate, dehydrated in a graded series of ethanol, and finally embedded in Epon resin. Ultrathin (60- to 70-nm-thick) sections, obtained with a Leica EM UC6 ultramicrotome, were collected on 200 mesh copper grids. The grids were observed in a Jeol JEM 1011 electron microscope operating at 100 kV and recorded with a 4-megapixel Gatan Orius SC100 charge-coupled device camera. To test for specificity of the immunocytochemical procedures, the HA antibody was omitted, or, alternatively, cells were transfected with an empty vector. Under either condition, no immunoreactivity was observed.

##### Post-embedding Immunogold Technique

Post-embedding immunogold labeling was performed 48 h after transfection. COS7 cells were detached from the Petri dish and spun at 1500 rpm. The pellet was fixed with 2% paraformaldehyde and 0.2% glutaraldehyde in 0.1 m phosphate buffer (pH 7.4), stained with 0.5% uranyl acetate, dehydrated, and finally embedded in Lowicryl HM20 under UV light for 2 days. Ultrathin sections (60- to 70-nm thick) were collected on formvar-coated nickel grids. Then post-embedding immunolabeling was performed. Briefly, sections were washed with 0.1% sodium borohydride and 50 mm glycine and incubated with the primary antibody. The samples were subsequently washed with TBS/0.1% Triton X-100 and incubated with goat anti-rabbit IgG coupled to 10-nm gold particles diluted 1:100. Sections were then post-fixed in glutaraldehyde in TBS, washed with distilled water, and finally stained with uranyl acetate and lead citrate. The grids were observed in a Jeol JEM 1011 electron microscope as described above.

##### Cryo-immunogold Technique

COS7 cells were transiently co-transfected with both GFP-PRRT2 and PRRT2-HA and embedded in 2% gelatin in 0.1 m phosphate buffer. Ultrathin 40-nm sections were obtained with a Leica EM UC6 ultramicrotome using the Tokuyasu technique (29–30) and collected on formvar-coated nickel grids. Sections were then incubated with mouse anti-GFP (1:100, Millipore) and rabbit anti-HA (1:100, Bethyl Laboratories) primary antibodies. Samples were then incubated with goat anti-mouse and goat anti-rabbit secondary antibodies (1:100, Nanoprobes) conjugated to colloidal nanogold particles of 10 nm (GFP labeling) and 6 nm (HA labeling), respectively. The grids were observed in a Jeol JEM 1011 electron microscope as described above.

### SH3 Affinity Chromatography

These experiments were performed as described previously (27, 31–33). The eluted proteins were separated by 12% SDS-PAGE and analyzed by immunoblotting with anti-PRRT2 and anti-dynamin I antibodies.

### Co-immunoprecipitation Assays

For immunoprecipitation, 10 μg of mouse anti-Intersectin 1 antibodies (clone 29, BD Biosciences), goat anti-Endophilin 1 antibodies (catalog no. sc-10874, Santa Cruz Biotechnology), or mouse/goat control IgGs (Sigma-Aldrich) were precoated with protein G Sepharose (GE Healthcare) overnight and incubated with total mouse brain lysate in immunoprecipitation buffer (150 mm NaCl, 50 mm Tris-HCl (pH 7.4), 2 mm EDTA, and 1% Triton X-100). After extensive washes in immunoprecipitation buffer and detergent-free immunoprecipitation buffer, samples were resolved by SDS-PAGE and subjected to Western blotting with anti-PRRT2 antibodies.

### Structure Modeling

We used the Robetta web server (34) to model the structure of the PRRT2 transmembrane domain, providing as input sequence residues Gly-261 to Lys-340. Robetta implements parts of the Rosetta structure prediction software suite (35) to generate models of protein domains by automatically combining template-based homology modeling and *de novo* approaches. It first screens the sequence using BLAST, PSI-BLAST, and 3D-Jury for regions that possess a homolog with an experimentally determined structure and then parses the sequence into putative domains on the basis of matches to both known and predicted structures. Any remaining region is cut up into small sizes and modeled via the Rosetta *de novo* protocol. On the basis of a coarse-grained energy function, the lowest-energy models are selected and combined into a final full-length model by optimizing the interactions between domains. The best-scoring PRRT2 model showed good values for standard quality assessment parameters, normalized Qmean 0.49 and Z-score −1.85. Note that the average score of high-resolution x-ray structures is around 0 and that membrane proteins usually receive lower Z scores than soluble proteins (36).

### Molecular Dynamics Simulations

Molecular dynamics (MD) simulations were performed using NAMD2.9 (37) with the CHARMM27 force field for proteins (with cross-term energy map corrections), ions, and water and the CHARMM36 force field for lipids (38). The protein transmembrane domain (Gly-261 to Lys-340 of the human PRRT2 sequence, which is identical to the mouse sequence Gly-267 to Lys-346) was inserted into a pre-equilibrated 1-palmitoyl-2-oleoyl-*sn*-glycero-3-phosphocholine (POPC) lipid bilayer and solvated with water molecules and counterions to neutralize the total system charge. The total number of atoms in the system was about 63,000. Periodic boundary conditions were applied, and the particle mesh Ewald method was used for long-range electrostatics (39), with a grid spacing of 1 Å and sixth-order B-splines. A cutoff of 12 Å and smooth switching at 10 Å was used for Lennard-Jones interactions. Chemical bond distances involving hydrogen atoms were constrained using the SHAKE/RATTLE algorithm (40). The full system was energy minimized for 2000 steps, and then its temperature was increased up to 300 K over 1.2-ns MD simulation by a sequence of 100-ps-long simulations at constant pressure (1 atm) and temperature, increased by 25 K at each stage. The system was equilibrated by running 1-ns NPT simulation with *p* = 1 atm and *T* = 310 K with the Langevin piston Nosé-Hoover method (41). The simulation ensemble was then switched to a constant volume and temperature (NVT) by keeping the temperature stationary around 310 K via Langevin dynamics with a damping coefficient 5 ps^−1^ and time step of 1 fs. Two independent simulations lasting 20 and 50 ns were generated using this protocol.

### Statistical Analysis

Data are expressed as mean ± S.E. throughout. To compare two normally distributed sample groups, two-tailed Student's *t* test was used. In the case of more than two normally distributed experimental groups, one-way analysis of variance followed by multiple comparison tests was employed. The significance level was preset to *p* < 0.05. Data were analyzed using the Prism 6.0 and SigmaPlot 10.0 softwares.

## Results

### 

#### 

##### Live Labeling Defines a Novel Membrane Topology for PRRT2

The predicted membrane topology of the Dispanin family consists of two transmembrane domains connected by a short intracellular loop with both the N-terminal and C-terminal domains oriented toward the outside of the cell. To verify whether PRRT2 shares this topology, we generated three distinct constructs bearing a triplet of HA tags alternatively at the N terminus (HA-PRRT2), at the C terminus (PRRT2-HA), or within the loop connecting the two putative transmembrane segments of PRRT2 (PRRT2-loop-HA).

**FIGURE 1.**
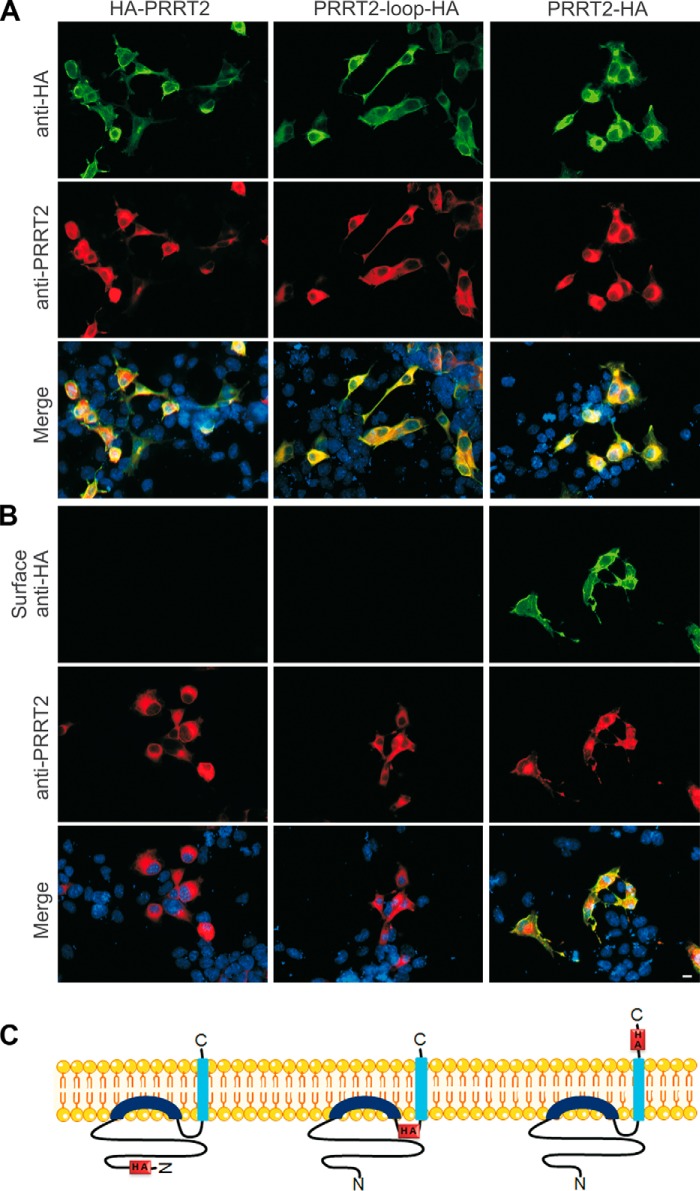
**Different localization of HA epitopes tagging the N and C termini of PRRT2 with respect to the plasma membrane.** *A*, COS7 cells were transfected with distinct HA-tagged PRRT2 constructs (HA-PRRT2, with HA_3_ tags at the N terminus; PRRT2-loop-HA, with HA_3_ tags between the two hydrophobic segments in the predicted cytosolic domain; and PRRT2-HA, with HA_3_ tags at the C terminus), fixed, and permeabilized for standard immunofluorescence. After permeabilization, both anti-HA (*top panels*) and anti-PRRT2 (*center panels*) antibodies recognized the overexpressed protein to the same extent (*Merge*, *bottom panels*). Cell nuclei were counterstained with DAPI (*blue*). *B*, live labeling with anti-HA antibodies only recognized PRRT2-HA, and no signal was detected in cells transfected with PRRT2-loop-HA or HA-PRRT2, indicating that the HA epitopes of these constructs were not accessible on the external surface of the cells (*top panels*). The different HA constructs were expressed equally, as shown by immunostaining with anti PRRT2 antibodies obtained after permeabilization of the cells (*center panels*). *Bottom panels*, merged images from the *top* and *center panels. Scale bar* = 20 μm. *C*, schematic of the membrane topology of PRRT2. The orientation of the N and C termini of PRRT2 with respect to the plasma membrane is shown, with the position of the HA_3_ tags in the protein sequence of the three constructs used to determine the topology of the protein.

When cells transfected with the various PRRT2 constructs were permeabilized and labeled with anti-HA antibodies, all three HA isoforms were detected with comparable expression levels (Fig. 1*A*, *top panels*). Anti-PRRT2 antibodies, directed to an N-terminal epitope of PRRT2, also recognized the three isoforms to the same extent (Fig. 1*A*, *center panels*) and, as expected, the labeling pattern overlapped completely with the HA staining (Fig. 1*A*, *bottom panels*). In contrast, when cells were live-labeled, maintaining the integrity of the plasma membrane, HA antibodies exclusively recognized the PRRT2-HA isoform, confirming exposure of the C terminus of PRRT2 to the extracellular surface. Moreover, no signal could be detected in live cells transfected with PRRT2-loop-HA, confirming that this putative cytosolic domain is indeed intracellular. Strikingly, HA antibodies did not recognize any exposure of HA-PRRT2 to the extracellular environment, indicating that the long N-terminal domain of PRRT2 is not exposed on the outer surface of the cell but, rather, is intracellular (Fig. 1*B*, *top panels*). After permeabilization, PRRT2 labeling was clearly visible to the same extent in cells transfected with any of the three constructs (Fig. 1*B*, *center panels*), showing that the three constructs had a similar transfection efficiency and that the HA_3_ tag fused to the C terminus did not affect the membrane insertion of PRRT2. PRRT2 staining overlapped with live HA labeling only in cells transfected with PRRT2-HA (Fig. 1*B*, *bottom panels*). These results suggest a new topology for PRRT2, as schematized in Fig. 1*C*.

**FIGURE 2.**
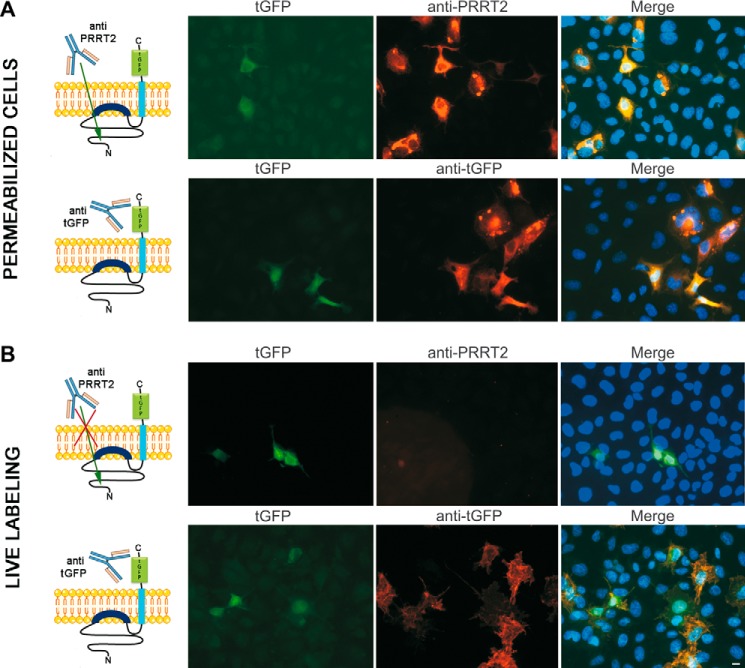
**Tagging PRRT2 with an alternative fluorescent epitope confirms the opposite location of the N and C termini with respect to the plasma membrane.** *A*, COS7 cells were transfected with a plasmid encoding for PRRT2 with C-terminal tGFP. After permeabilization, both anti-PRRT2 antibodies recognizing the N terminus (sequence 152–268) and anti-tGFP antibodies detected the transfected protein. *B*, live labeling with the same antibodies was present only with anti-tGFP antibodies, suggesting that the N-terminal epitopes are not exposed on the external side of the plasma membrane. *Scale bar* = 20 μm. The different accessibility of PRRT2 epitopes to the antibodies used in each experiment is shown in the *left panels*. When cells are permeabilized, anti-PRRT2 antibodies can enter the cells and recognize intracellular N-terminal epitopes. Under live labeling condition, the intact plasma membrane blocks their accessibility to N-terminal epitopes.

Additional evidence of this novel topology of PRRT2 was obtained by transfecting COS7 cells with a plasmid encoding for PRRT2 fused at the C-terminal with a distinct fluorescent tag, turbo-GFP (PRRT2-tGFP), and analyzing the cells with a combination of anti-PRRT2 and anti-tGFP antibodies under both permeabilizing and non-permeabilizing conditions. The PRRT2-tGFP chimera was well expressed in cells, as shown by the intrinsic tGFP fluorescence (Fig. 2, *A* and *B*). When cells were permeabilized, both anti-PRRT2 antibodies (recognizing the sequence 152–268 in the N terminus of the protein) and anti-tGFP antibodies detected the PRRT2-tGFP fusion protein (Fig. 2*A*, *red signals*). However, under live labeling conditions, only the tGFP antibodies detected the C-terminal tGFP, and no signal was detected by anti-PRRT2 antibodies, further confirming, with distinct constructs and combination of antibodies, that the N-terminal epitopes are not accessible when the plasma membrane is intact in the absence of detergents and fixatives (Fig. 2*B*, *red signal*). The different accessibility of PRRT2 epitopes to the antibodies used is schematized in the *left panels* of Fig. 2. Taken together, these results suggest a topology for the PRRT2 protein that is clearly distinct from that predicted on the basis of homology with the Dispanin family and consistent with a type II transmembrane protein characterized by an N_cyt_/C_exo_ orientation, whereby only the second hydrophobic segment spans the plasma membrane, the first hydrophobic segment is associated putatively with the interior of the plasma membrane, and the N terminus of the protein is intracellular (Fig. 1*C*).

##### Immunogold Labeling Visualizes the C-terminal and N-terminal Domains of PRRT2 on Opposite Sides of the Plasma Membrane

**FIGURE 3.**
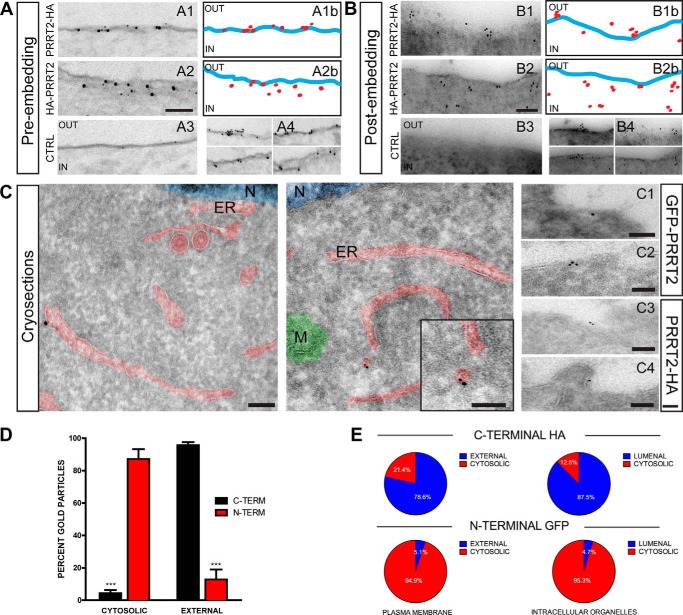
**Ultrastructural localization of the C and N termini of PRRT2 expressed in COS7 cells.** *A* and *B*, representative micrographs, obtained with the pre-embedding (*A*) or post-embedding (*B*) technique, of COS7 cells transfected with either C-terminal PRRT2-HA (*A1* and *B1*) or N-terminal HA-PRRT2 (*A2* and *B2*) and immunogold-labeled with anti-HA antibodies. Non-transfected cells (*CTRL*, *A3* and *B3*) were also processed as negative controls. *A1b* and *A2b*, ImageJ masks of *A1* and *A2*, highlighting the plasma membrane (*blue*) and the gold particles (*red*) mapping the N and C termini of PRRT2. *B1b* and *B2b*, ImageJ masks of *B1* and *B2*, highlighting the plasma membrane (*blue*) and the gold particles (*red*). *A4* and *B4*, additional images of plasma membrane domains of transfected COS7 cells mapping the C-terminal (*top panels*) or N-terminal PRRT2 (*bottom panels*). *C*, representative EM micrographs of PRRT2-expressing COS7 cells obtained with the Tokuyasu cryo-immunogold technique. Cells were co-transfected with N-terminally labeled GFP-PRRT2 and C-terminally labeled PRRT2-HA or left untransfected as a negative control. Representative cryosections labeled with anti-GFP antibodies coupled to 10-nm nanogold particles (*left panel*) and anti-HA antibodies coupled with 6-nm gold particles (*center panel* and *inset*) are shown. Additional images visualizing the location of GFP (*C1* and *C2*) and HA (*C3* and *C4*) immunoreactivities are also shown. *N*, nucleus; *ER*, endoplasmic reticulum; *M*, mitochondrion. *Scale bars* = 100 nm. *D*, quantification of the percentage of gold particles located inside (*cytosolic*) or outside of (*external*) the plasma membrane from pre- and post-embedding specimens. *Error bars* represent the mean ± S.E. (*n* = 46 images from 3 independent experiments) for the C-terminal (*black*) and N-terminal (*red*) labeling of PRRT2, respectively. ***, *p* < 0.01; Student's *t* test. *E*, the occurrence of gold particles decorating the C terminus (*C-terminal HA*) or the N terminus (*N-terminal GFP*) of PRRT2 calculated from the cryo-immunolabeled sections shown in *C*. The position of the gold particles was quantified with respect to both the plasma membrane (*cytosolic/external*) and the membrane of intracellular organelles (*cytosolic/lumenal*).

To analyze the localization of the N-terminal and C-terminal domains of PRRT2 in more detail, ultrathin sections (60–70 nm thick) of COS7 cells transfected with either HA-PRRT2 or PRRT2-HA constructs were immunogold-labeled for the HA tag and analyzed by electron microscopy using both pre-embedding and post-embedding techniques. With both techniques, when cells were transfected with C-terminally tagged PRRT2-HA and incubated with the primary anti-HA antibodies, gold particles were localized specifically to the extracellular side of the plasma membrane (Fig. 3*A1*, *A4* and *B1*, *B4*, *top panels*). In contrast, when cells were transfected with the N-terminally tagged HA-PRRT2, gold particles were only present on the cytosolic side of the plasma membrane, consistent with an intracellular location of the N terminus of the protein (Fig. 3, *A2*, *A4* and *B2*, *B4*, *bottom panels*). The topography of the immunogold particles with respect to the plasma membrane is shown schematically in the ImageJ masks of the immuno-EM images (Fig. 3, *A1b*, *A2b* and *B1b*, *B2b*). When the primary anti-HA antibody was omitted or the cells were not transfected with the PRRT2 constructs, no immunoreactivity was observed (Fig. 3, *A3* and *B3*), confirming the specificity of the immunolabeling. Quantification of the immunolabeling obtained with both techniques showed that the C terminus was almost entirely located extracellularly, whereas the vast majority of the staining for the N terminus was cytosolic (Fig. 3*D*). In some sections, few gold particles were visualized inside the cell with both N-terminal and C-terminal domain staining of PRRT2. These intracellular pools presumably reflect PRRT2 trafficking in the biosynthetic and secretory pathways.

To further confirm the above data and obtain detailed mapping of PRRT2 across the internal membranes, such as those of the endoplasmic reticulum, that were not well preserved using the pre- and post-embedding techniques, we used the cryo-dual immunogold technique in cells co-expressing both N-terminal GFP and C-terminal HA tags. Cryosections were labeled with anti-GFP antibodies coupled to 10-nm nanogold particles and anti-HA antibodies coupled to 6-nm gold particles (Fig. 3*C*). The data of the dual labeling fully confirmed our previous results, showing the localization of the C terminus of PRRT2 both outside the plasma membrane and inside the intracellular organelles, whereas the localization of the N terminus was cytosolic (Fig. 3*E*).

##### PRRT2 Is Surface-biotinylated at the C-terminal Domain

**FIGURE 4.**
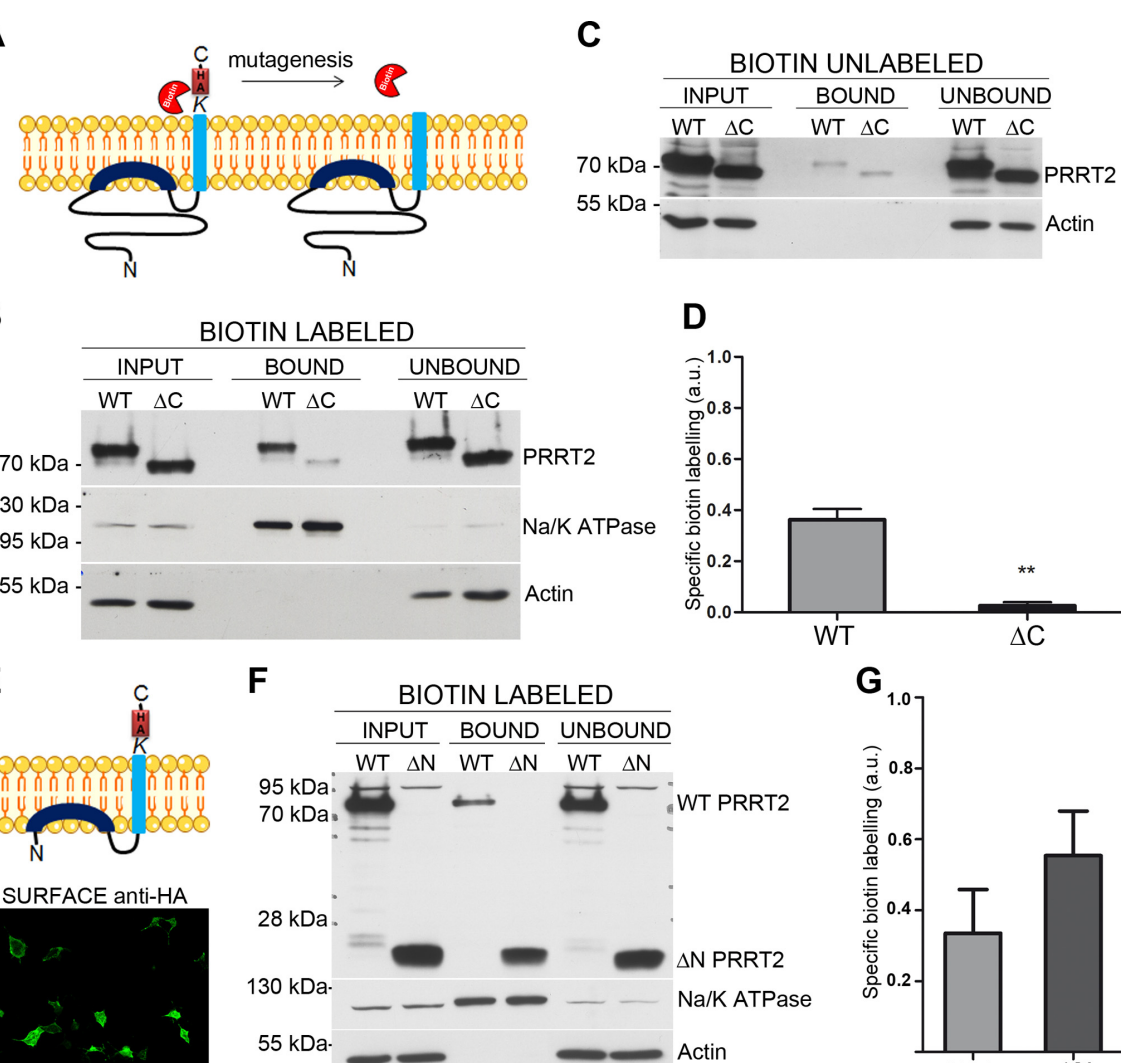
**PRRT2 is surface-biotinylated at the C-terminal domain.** *A*, biotin labeling of the C-terminal lysine residue of PRRT2 and the lack of labeling of the deletion mutant PRRT2ΔC lacking the C-terminal domain and the HA tag. *B*, Western blotting analyses probed with anti-PRRT2 antibodies showed biotin labeling of wild-type PRRT2-HA and a strong decrease in biotinylation of PRRT2ΔC. Both isoforms were expressed equally in the cells (*input*). Their presence in the unbound fraction corresponds to the intracellular pool of the overexpressed protein. Na^+^/K^+^ ATPase and actin were used as positive and negative controls of surface biotinylation, respectively. *C*, representative control experiment in which biotin was omitted. The faint PRRT2 bands represent a low degree of nonspecific binding to the NeutrAvidin beads. *D*, immunoblots were quantified by densitometric analysis of the fluorograms obtained in the linear range of the emulsion response. The specific biotin labeling of PRRT2 was calculated as [total biotinylation/input − nonspecific biotinylation/input] and is expressed in arbitrary units (*a. u.*). Specific biotinylation, shown as mean ± S.E. of three independent experiments, is decreased significantly in PRRT2ΔC compared with WT PRRT2-HA. **, *p* < 0.01; Student's *t* test for paired samples. *E*, the PRRT2 mutant lacking the N-terminal domain (PRRT2ΔN) and its plasma membrane expression by live labeling with anti HA antibodies. *F* and *G*, representative Western blotting analysis probed with anti-HA antibodies (*F*) and quantification of the specific biotinylation (*G*, mean ± S.E., *n* = 3) showing that both WT PRRT2-HA and PRRT2ΔN are biotinylated specifically to approximately the same extent.

To get additional evidence supporting the new emerging topology of PRRT2, we performed protein surface biotinylation experiments, a useful method to identify the extracellular domains of membrane proteins. When the primary structure of mouse PRRT2 was analyzed, of the 12 lysines representing the preferential biotinylation sites, 10 were present in the N-terminal domain, one in the intracellular loop (Lys-320), and one in the C-terminal domain as the last amino acid of the protein sequence (Lys-346). If the novel PRRT2 topology suggested by the above-described results were true, then the C-terminal lysine would be the only residue accessible to extracellular biotin labeling, whereas the multiple N-terminal lysines would be intracellular and could not bind biotin. To demonstrate the crucial role of the C-terminal lysine in biotinylation experiments, we generated a PRRT2 mutant by inserting a stop codon after valine 344 in the PRRT2-HA construct. This PRRT2 mutant, lacking the C-terminal lysine (and the C-terminal HA tag), is predicted not to bind biotin at the C terminus of the protein (PRRT2ΔC, Fig. 4*A*).

COS7 cells were transfected with either wild-type PRRT2-HA or PRRT2ΔC, incubated with the biotin reagent, precipitated with NeutrAvidin beads, and analyzed by Western blotting. PRRT2 was present in the biotinylated fraction, suggesting that at least one domain of the protein was extracellular and bound biotin. Interestingly, the mutation of the C-terminal lysine virtually abolished biotinylation of the protein, suggesting that this residue was indeed responsible for the surface labeling (Fig. 4*B*, *top*). The residual faint band that was still visible in the bound fraction with the C-terminal mutant of PRRT2 represents nonspecific binding of the protein to the beads because it was still present when the biotin reagent was omitted (Fig. 4*C*). Quantification of the immunoblots confirmed the strong decrease in specific biotin labeling of the PRRT2ΔC isoform *versus* the WT (Fig. 4*D*).

**FIGURE 5.**
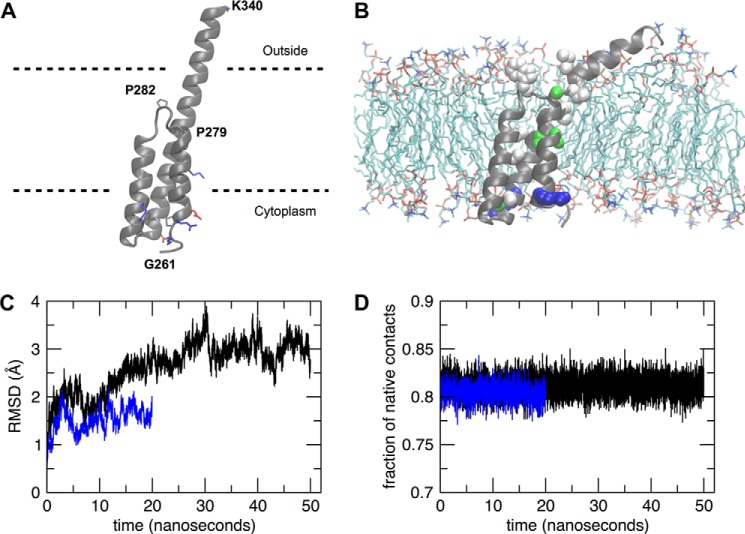
**Molecular dynamics simulations of PRRT2 topology.** *A*, predicted model of the PRRT2 protein transmembrane domains (residues 261–340). The protein backbone is shown as a *schematic*, whereas charged residues are shown as *sticks* and colored according to their type (*blue*, basic; *red*, acidic). Proline residues are also shown as *sticks. Dashed lines* indicate putative membrane limits. *B*, snapshot of the protein and membrane system at the end of the 20-ns MD trajectory. Water molecules and some lipids were removed for clarity. The residues mutated in the PRRT2 sequence (6) are shown as *spheres* and colored according to their type (*blue*/*red*, charged; *green*, polar; *white*, hydrophobic). *C*, root mean square displacement (*RMSD*) of the protein structure with respect to the starting conformation along the 20-ns (*blue*) and 50-ns (*black*) MD trajectories. *D*, fraction of native contacts along the 20-ns (*blue*) and 50-ns (*black*) MD trajectories.

To confirm that the N-terminal lysines are not involved in biotinylation, we generated an additional PRRT2 mutant lacking the N-terminal domain (PRRT2ΔN, Fig. 4*E*). The construct encodes PRRT2 residues from Gly-267 to Lys-346 (the very same sequence modeled by MD simulations, see Fig. 5) plus the HA_3_ tag, and lacks almost completely the N-terminal domain together with the nine N-terminal lysines. Moreover, we additionally mutated Lys-270 into an arginine (similarly charged residue but not prone to biotinylation) so that the PRRT2ΔN mutant retained only two lysines: Lys-320 between the two putative transmembrane domains and the C-terminal Lys-346. Despite the large deletion, the PRRT2ΔN mutant was exposed correctly to the plasma membrane, as shown by live labeling with anti HA antibodies (Fig. 4*E*). Biotinylation assays of PRRT2ΔN showed that this mutant was biotinylated effectively to the same extent as PRRT2-HA, confirming that the lysine residue responsible for PRRT2 biotinylation is Lys-346 and that the N-terminal lysines are not involved in the reaction (Fig. 4, *F* and *G*).

To check the reliability of surface biotinylation and the integrity of the plasma membrane, we analyzed intracellular proteins such as actin. Indeed, actin was totally absent from the biotinylated fraction and was only present in the unbound fraction (Fig. 4, *B–E*, and *F*, *bottom panel*). Moreover, a positive control of biotinylation, *i.e.* the transmembrane protein Na^+^/K^+^-ATPase, was enriched, as expected, in the biotinylated fraction (Fig. 4 *B–E*, and *F*, *center panel*). Both WT and mutant PRRT2 isoforms were also present in the NeutrAvidin-unbound fraction, representing an intracellular pool of the protein being processed by the secretory machinery of the cell. These results confirm, with a fully independent approach, the extracellular exposure of the C terminus and the intracellular localization of the Lys-rich N-terminal region of PRRT2.

##### Structural Modeling and MD Simulations of PRRT2 Topology

All-atom structural models of the PRRT2 protein transmembrane domain, including residues Gly-261 to Lys-340 of human PRRT2, were generated using the Rosetta online software (35). Among the obtained models, the best-scoring one (Fig. 5*A*) has good structural quality assessment values, a normalized Qmean of 0.49, and a Z score of −1.85 (note that membrane proteins usually get lower Z scores than soluble proteins (34)). In the model, charged residues are located in two distant regions of the chain, interspersed by mostly hydrophobic amino acids. This clearly identifies putative solvent-accessible and membrane-spanning regions of the chain. The most striking feature of the structure is the helix-loop-helix motif displayed by the TM1 domain (Ile-269 to Ala-289), with two α helices extending from residues Ile-269 to Pro-279 and from residues Ile-285 to Ala-289, linked by a short hinge comprising residues Met-280 to Asn-284. This particular conformation keeps both edges of the TM1 segment in the cytoplasm, implying that the model is consistent with our hypothesis that the full PRRT2 protein N-terminal is in the cytoplasmic region. Interestingly, the hinge occurs in the proximity of two proline residues (Pro-279 and Pro-282). The region Tyr-290 to Ser-317, connecting TM1 to TM2 and referred to previously as the cytoplasmic loop, is indeed on the same side of the N-terminal and, therefore, also in the cytoplasm, although it shows some degree of secondary structure.

To further validate and refine the PRRT2 transmembrane segment model, we performed all-atom MD simulations of this protein portion in a water-membrane model environment (POPC lipids). To insert the protein in the membrane, we based ourselves on the location of hydrophobic and charged amino acids, taking care that most of the charged residues were exposed to the solvent and that the hydrophobic ones were within the membrane. We performed two independent simulations, one lasting 20 ns and one 50 ns, starting from two slightly different protein-membrane conformations. A snapshot of the simulated protein and membrane system at the end of the 20-ns trajectory is shown in Fig. 5*B*, where water and some lipid molecules were removed for clarity. In the figure, residues belonging to a list of PRRT2 point mutations (6) are shown as spheres colored according to their type (*blue*, basic; *red*, acidic; *green*, polar; *white*, hydrophobic). The structure was overall stable along both simulations, showing a root mean square displacement from the starting conformation that reached plateaus around 1.5 and 3 Å for the 20- and 50-ns trajectories, respectively (Fig. 5*C*). The fraction of native contacts was stationary along both simulated trajectories (Fig. 5*D*), again revealing a stable structure.

##### The Intracellular Proline-rich N-terminal Domain Binds SH3 Domains

**FIGURE 6.**
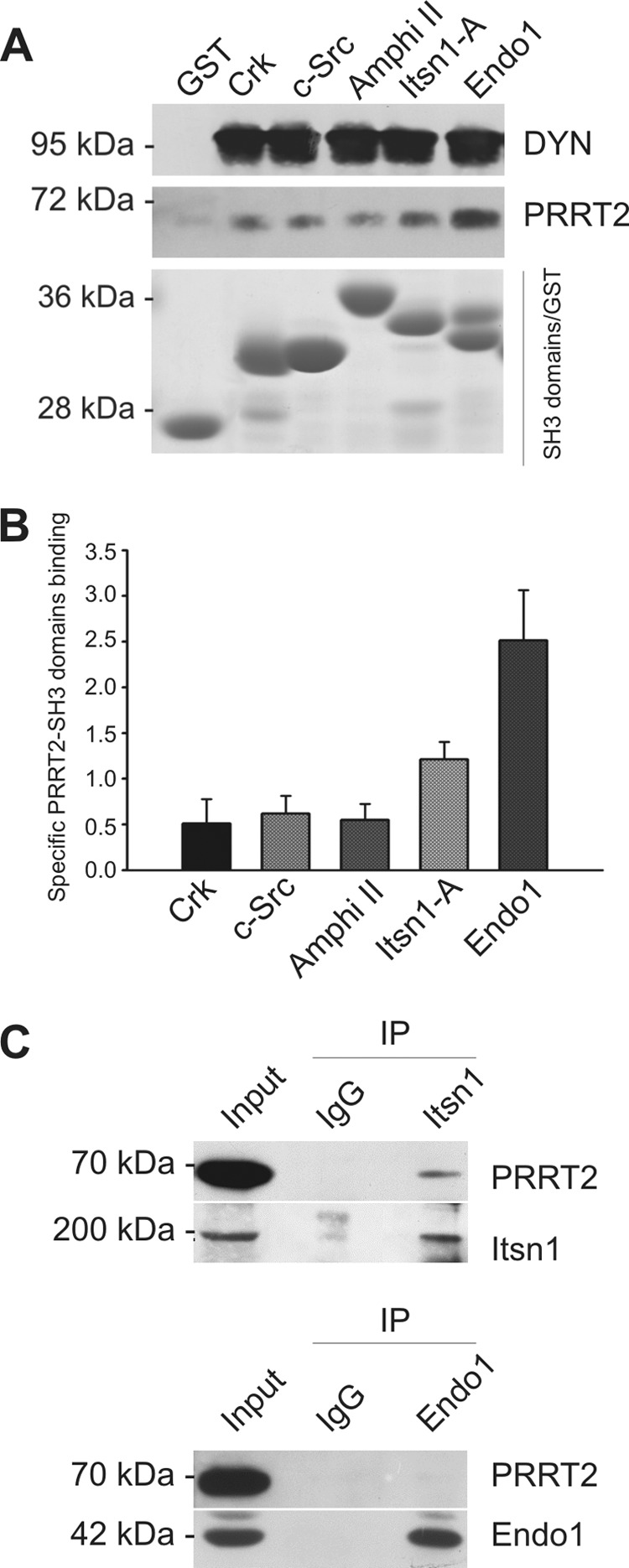
**Affinity purification of PRRT2 by SH3 domains from extracts of brain synaptosomes** *A*, pulldown assay. Affinity resins were prepared by coupling the SH3 domains of Crk, c-Src, Amphiphysin II (*Amphi II*), Intersectin 1 SH3-A (*Itsn1-A*), or Endophilin 1 (*Endo1*) or GST alone to glutathione-Sepharose. Columns were loaded with a Triton X-100 extract of crude synaptosomal fraction (P2) from rat forebrain. Bound proteins were eluted with SDS, separated by SDS-PAGE, and analyzed by immunoblotting with anti-PRRT2- or anti-dynamin (*DYN*)-specific antibodies. *Center panel*, the elution patterns of PRRT2. The immunoreactivity of dynamin I (*top panel*) is shown for comparison. *Bottom panel*, recovery of the GST-tagged SH3 domains after Coomassie staining of the gels. *B*, quantification of the pulldown. Immunoblots were analyzed by densitometry of the fluorograms obtained in the linear range of the emulsion response to quantify band immunoreactivity. The amount of PRRT2 associated with the respective SH3 domains was calculated as the ratio of specific PRRT2-SH3 domain binding (immunoreactivity of the PRRT2 SH3 GST domain − immunoreactivity of PRRT2 GST) with the unspecific binding (immunoreactivity of PRRT2 GST) and expressed as mean ± S.E. (*n* = 5). *C*, co-immunoprecipitation. Detergent extracts of mouse brain were immunoprecipitated with anti-intersectin 1 monoclonal antibodies, anti-endophilin-1 monoclonal antibodies, or mouse IgG, as indicated (*IP*). After electrophoretic separation of the immunocomplexes and Western blotting, membranes were probed with antibodies against Itsn1 and Endo1 to test the efficiency of the immunoprecipitation (*bottom blots*) and with anti-PRRT2 antibodies (*top blots*).

Several minimal Pro-*X-X*-Pro consensus sequences for Src homology 3 (SH3) domain binding are present in the primary structure of the N-terminal domain of PRRT2 (proline-rich domain; residues 133–136, 157–160, 206–209, and 242–245 in the N-terminal domain of mouse PRRT2). After we established that this domain is exposed to the interior of the cell, we preliminarily assessed whether it is able to bind SH3 domains from an array of proteins involved in signal transduction and/or synaptic vesicle cycling. Therefore, we asked whether PRRT2 could be specifically affinity-purified from an extract of crude synaptosomal fractions (P2) obtained from rat forebrain by the SH3 domains of Crk, c-Src, Amphiphysin II, Intersectin 1 SH3-A, and Endophilin 1, expressed as fusion proteins with GST, using GST alone as nonspecific binding control. PRRT2 was pulled down to various extents by the individual SH3 domains. Notably, the Endophilin 1 and Intersectin 1 SH3-A domain displayed the highest interactions (Fig. 6, *A* and *B*). As an internal control, all SH3 domains analyzed also bound dynamin I to the same extent, as described previously (27). To verify whether these interactions with isolated recombinant SH3 domains could translate into an interaction with the holoprotein, we performed co-immunoprecipitation experiments with Endophilin 1 and Intersectin 1, two nerve terminal proteins implicated in the recycling of synaptic vesicles whose SH3 domain showed strong PRRT2 binding in the pulldown assay. Interestingly, anti-Intersectin 1 antibodies did co-immunoprecipitate PRRT2, whereas anti-Endophilin 1 antibodies failed to do so (Fig. 6*C*).

## Discussion

Understanding the topology of membrane proteins is an important step for the comprehension of the molecular mechanisms of protein function. PRRT2 is a protein with a putative role at the synapse that is responsible, when mutated, for various paroxysmal disorders of infancy. It is therefore of great importance to obtain clues regarding its function to understand the pathogenesis of these diseases. PRRT2 belongs to the family of Dispanin, whose putative topology has been predicted to consist of two transmembrane domains connected by a short intracellular loop and two extracellular N- and C-terminal domains. To unambiguously define the function of PRRT2 at the synapse, it is important to confirm the exact orientation of its N and C termini with respect to the plasma membrane to obtain clues regarding its possible key interactors and functions.

The experiments presented in this paper demonstrate unequivocally a novel PRRT2 topology by several experimental approaches. Live-cell labeling experiments, performed with various sets of PRRT2 constructs, showed identical results. The use of the same anti-HA antibodies to target the N and C termini of PRRT2 excludes possible artifacts due to antibody malfunction under live labeling conditions. When cells were not permeabilized, the N-terminal epitopes were not accessible by antibodies that, instead, recognized the C-terminal epitopes, indicating that the C terminus is, as predicted, exposed at the external surface of the cell, whereas the N terminus is inside of the cell, at variance with the predicted topology.

These results were confirmed by immunoelectron microscopy, which showed unambiguously the presence of C terminus immunoreactivity on the external leaflet of the plasma membrane, whereas N terminus immunoreactivity was confined inside the cell. The novel topology was also confirmed independently by surface biotinylation. Although the mutation of the single C-terminal lysine virtually abolished biotin labeling, the lack of 10 N-terminal lysines did not influence the reaction, suggesting that these residues are located intracellularly.

The new orientation of the N-terminal domain toward the intracellular space leads to the consequence that only the second C-terminal hydrophobic segment spans the plasma membrane and protrudes into the extracellular space with the short C-terminal domain. Therefore, the first N-terminal hydrophobic segment does not cross the membrane and remains putatively associated with its cytoplasmic leaflet. The structure of the C-terminal region was modeled computationally and refined further by MD simulations in a water-membrane environment. Through this advanced bioinformatics analysis, it was possible to ascertain that the α helix of TM1 flexes in correspondence with a five-amino acid hinge that includes two proline residues and forms a helix-loop-helix structure. Although helix destabilization by prolines is most frequently observed in soluble proteins, proline-induced distortions are also present in structures of transmembrane chains, where they are supposed to facilitate helical packing motifs (42). Therefore, both edges of the TM1 α helix protrude in the cytoplasm, where they are connected to the N-terminal domain and the cytoplasmic loop.

Taken together, these results define an alternative N_cyt_/C_exo_ PRRT2 topology consistent with a type II transmembrane protein in which only the second hydrophobic segment completely spans the plasma membrane, the first hydrophobic segment is associated with the internal half of the plasma membrane via a helix-loop-helix structure, and the N terminus is intracellular. PRRT2 is not the only member of the dispanin family to show a different topology with respect to the originally predicted one. The recently clarified topology of interferon-induced transmembrane proteins, constituting a subfamily of the dispanins, supports the novel PRRT2 topology demonstrated here. Recent reports have demonstrated that two members of this family, interferon-inducible transmembrane proteins 1 (28) and 3 (43), display a PRRT2-like topology, with the N terminus oriented toward the cytosolic compartment and the C terminus extracellular.

The novel PRRT2 topology demonstrated in this paper has important implications for PPRT2 function. In fact, the intracellular location of the N-terminal domain, which accounts for 80% of the primary structure of the protein, opens the possibility of novel interactions with intracellular proteins involved in synaptic function and leads to the formulation of completely different functional hypotheses for PRRT2. Interestingly, by means of pulldown assays, we showed binding of PRRT2 with a few SH3 domains belonging to synaptic and regulatory proteins involved in exo/endocytosis of synaptic vesicles or in signal transduction, including those of Endophilin 1 and Intersectin 1, which are key players in clathrin-mediated endocytosis at nerve terminals (44). Notably, co-immunoprecipitation assays confirmed an interaction of PRRT2 with Intersectin 1 but not with Endophilin 1. Although this discrepancy may result from differences in binding to an isolated SH3 domain or to the entire protein, the confirmed interaction with Intersectin 1 is additional proof of the intracellular localization of the PRRT2 N terminus and suggests a potential functional role of the protein in the regulation of vesicle trafficking and endocytosis.

The novel N_cyt_/C_exo_ topology of PRRT2 makes the demonstrated interaction of PRRT2 with the SNARE protein SNAP-25 more plausible. Although, according to the originally predicted PRRT2 topology, the interaction was confined to the single short cytosolic loop, the new PRRT2 topology with the large PRRT2 N-terminal domain inside the cell allows multiple interactions sites not only for SNAP-25 but also for other members of the presynaptic interactome. Most of the proteins playing key roles in the final priming/fusion steps of release, including synaptotagmins, vesicle-associated membrane protein, syntaxin, and SNAP-25 have large interacting cytosolic domains, and, notably, PRRT2, vesicle-associated membrane protein 2, and syntaxin are all type II transmembrane proteins with a C-terminal anchor. The novel topology of PRRT2 will give breathing space to the search for new intracellular interactors and will contribute to clarify the role of this important protein in synaptic transmission as well as the pathogenic mechanisms by which its mutations lead to neurological manifestations.

## Author Contributions

P. R. and B. S. performed the cell and molecular biology studies, including immunocytochemistry and surface biotinylation experiments. E. C. performed and analyzed the immunoelectron microscopy experiments. L. M. performed structure modeling studies and MD simulations. F. O. and A. M. performed the pulldown assays with SH3 domains. A. C., F. V., and F. B. designed and supervised the research and analyzed the data. F. B. and A. C. wrote the paper and supported the study.

## Footnotes

3 The abbreviations used are:

TM: transmembrane
MD: molecular dynamics
tGFP: turbo GFP
SH: Src homology.

## References

[1] Méneret A., Gaudebout C., Riant F., Vidailhet M., Depienne C., and Roze E. (2013) PRRT2 mutations and paroxysmal disorders. Eur. J. Neurol. 20, 872–878 23398397 10.1111/ene.12104

[2] Heron S. E., and Dibbens L. M. (2013) Role of PRRT2 in common paroxysmal neurological disorders: a gene with remarkable pleiotropy. J. Med. Genet. 50, 133–139 23343561 10.1136/jmedgenet-2012-101406

[3] Becker F., Schubert J., Striano P., Anttonen A. K., Liukkonen E., Gaily E., Gerloff C., Müller S., Heußinger N., Kellinghaus C., Robbiano A., Polvi A., Zittel S., von Oertzen T. J., Rostasy K., Schöls L., Warner T., Münchau A., Lehesjoki A. E., Zara F., Lerche H., and Weber Y. G. (2013) PRRT2-related disorders: further PKD and ICCA cases and review of the literature. J. Neurol. 260, 1234–1244 23299620 10.1007/s00415-012-6777-y

[4] Guerrini R., and Mink J. W. (2012) Paroxysmal disorders associated with PRRT2 mutations shake up expectations on ion channel genes. Neurology 79, 2086–2088 23077020 10.1212/WNL.0b013e3182752edd

[5] Wood H. (2012) Genetics: Expanding the spectrum of neurological disorders associated with PRRT2 mutations. Nat. Rev. Neurol. 8, 657 23165339 10.1038/nrneurol.2012.240

[6] Lee H. Y., Huang Y., Bruneau N., Roll P., Roberson E. D., Hermann M., Quinn E., Maas J., Edwards R., Ashizawa T., Baykan B., Bhatia K., Bressman S., Bruno M. K., Brunt E. R., Caraballo R., Echenne B., Fejerman N., Frucht S., Gurnett C. A., Hirsch E., Houlden H., Jankovic J., Lee W. L., Lynch D. R., Mohammed S., Müller U., Nespeca M. P., Renner D., Rochette J., Rudolf G., Saiki S., Soong B. W., Swoboda K. J., Tucker S., Wood N., Hanna M., Bowcock A. M., Szepetowski P., Fu Y. H., and Ptáček L. J. (2012) Mutations in the gene PRRT2 cause paroxysmal kinesigenic dyskinesia with infantile convulsions. Cell Rep. 1, 2–12 22832103 10.1016/j.celrep.2011.11.001 PMC3334308

[7] Sällman Almén M., Bringeland N., Fredriksson R., and Schiöth H. B. (2012) The dispanins: a novel gene family of ancient origin that contains 14 human members. PLoS ONE 7, e31961 22363774 10.1371/journal.pone.0031961 PMC3282796

[8] Chen W. J., Lin Y., Xiong Z. Q., Wei W., Ni W., Tan G. H., Guo S. L., He J., Chen Y. F., Zhang Q. J., Li H. F., Lin Y., Murong S. X., Xu J., Wang N., and Wu Z. Y. (2011) Exome sequencing identifies truncating mutations in PRRT2 that cause paroxysmal kinesigenic dyskinesia. Nat. Genet. 43, 1252–1255 22101681 10.1038/ng.1008

[9] Heron S. E., Grinton B. E., Kivity S., Afawi Z., Zuberi S. M., Hughes J. N., Pridmore C., Hodgson B. L., Iona X., Sadleir L. G., Pelekanos J., Herlenius E., Goldberg-Stern H., Bassan H., Haan E., Korczyn A. D., Gardner A. E., Corbett M. A., Gécz J., Thomas P. Q., Mulley J. C., Berkovic S. F., Scheffer I. E., and Dibbens L. M. (2012) PRRT2 mutations cause benign familial infantile epilepsy and infantile convulsions with choreoathetosis syndrome. Am. J. Hum. Genet. 90, 152–160 22243967 10.1016/j.ajhg.2011.12.003 PMC3257886

[10] Cloarec R., Bruneau N., Rudolf G., Massacrier A., Salmi M., Bataillard M., Boulay C., Caraballo R., Fejerman N., Genton P., Hirsch E., Hunter A., Lesca G., Motte J., Roubertie A., Sanlaville D., Wong S. W., Fu Y. H., Rochette J., Ptácek L. J., and Szepetowski P. (2012) PRRT2 links infantile convulsions and paroxysmal dyskinesia with migraine. Neurology 79, 2097–2103 23077017 10.1212/WNL.0b013e3182752c46 PMC3511924

[11] de Vries B., Callenbach P. M., Kamphorst J. T., Weller C. M., Koelewijn S. C., ten Houten R., de Coo I. F., Brouwer O. F., van den Maagdenberg A. M. (2012) PRRT2 mutation causes benign familial infantile convulsions. Neurology 79, 2154–2155 23077019 10.1212/WNL.0b013e3182752c30

[12] Gardiner A. R., Bhatia K. P., Stamelou M., Dale R. C., Kurian M. A., Schneider S. A., Wali G. M., Counihan T., Schapira A. H., Spacey S. D., Valente E. M., Silveira-Moriyama L., Teive H. A., Raskin S., Sander J. W., Lees A., Warner T., Kullmann D. M., Wood N. W., Hanna M., and Houlden H. (2012) PRRT2 gene mutations: from paroxysmal dyskinesia to episodic ataxia and hemiplegic migraine. Neurology 79, 2115–2121 23077024 10.1212/WNL.0b013e3182752c5a PMC3511930

[13] Marini C., Conti V., Mei D., Battaglia D., Lettori D., Losito E., Bruccini G., Tortorella G., and Guerrini R. (2012) PRRT2 mutations in familial infantile seizures, paroxysmal dyskinesia, and hemiplegic migraine. Neurology 79, 2109–2114 23077026 10.1212/WNL.0b013e3182752ca2 PMC3511926

[14] Riant F., Roze E., Barbance C., Méneret A., Guyant-Maréchal L., Lucas C., Sabouraud P., Trébuchon A., Depienne C., and Tournier-Lasserve E. (2012) PRRT2 mutations cause hemiplegic migraine. Neurology 79, 2122–2124 23077016 10.1212/WNL.0b013e3182752cb8

[15] Scheffer I. E., Grinton B. E., Heron S. E., Kivity S., Afawi Z., Iona X., Goldberg-Stern H., Kinali M., Andrews I., Guerrini R., Marini C., Sadleir L. G., Berkovic S. F., and Dibbens L. M. (2012) PRRT2 phenotypic spectrum includes sporadic and fever-related infantile seizures. Neurology 79, 2104–2108 23077018 10.1212/WNL.0b013e3182752c6c PMC3511925

[16] Wu L., Tang H. D., Huang X. J., Zheng L., Liu X. L., Wang T., Wang J. Y., Cao L., and Chen S. D. (2014) PRRT2 truncated mutations lead to nonsense-mediated mRNA decay in paroxysmal kinesigenic dyskinesia. Parkinsonism Relat. Disord. 20, 1399–1404 25457817 10.1016/j.parkreldis.2014.10.012

[17] Stelzl U., Worm U., Lalowski M., Haenig C., Brembeck F. H., Goehler H., Stroedicke M., Zenkner M., Schoenherr A., Koeppen S., Timm J., Mintzlaff S., Abraham C., Bock N., Kietzmann S., Goedde A., Toksöz E., Droege A., Krobitsch S., Korn B., Birchmeier W., Lehrach H., and Wanker E. E. (2005) A human protein-protein interaction network: a resource for annotating the proteome. Cell 122, 957–968 16169070 10.1016/j.cell.2005.08.029

[18] Li M., Niu F., Zhu X., Wu X., Shen N., Peng X., and Liu Y. (2015) PRRT2 Mutant leads to dysfunction of glutamate signaling. Int. J. Mol. Sci. 16, 9134–9151 25915028 10.3390/ijms16059134 PMC4463582

[19] Jahn R., and Fasshauer D. (2012) Molecular machines governing exocytosis of synaptic vesicles. Nature 490, 201–207 23060190 10.1038/nature11320 PMC4461657

[20] Schwenk J., Harmel N., Brechet A., Zolles G., Berkefeld H., Müller C. S., Bildl W., Baehrens D., Hüber B., Kulik A., Klöcker N., Schulte U., and Fakler B. (2012) High-resolution proteomics unravel architecture and molecular diversity of native AMPA receptor complexes. Neuron 74, 621–633 22632720 10.1016/j.neuron.2012.03.034

[21] Südhof T. C. (2008) Neuroligins and neurexins link synaptic function to cognitive disease. Nature 455, 903–911 18923512 10.1038/nature07456 PMC2673233

[22] Dityatev A., Schachner M., and Sonderegger P. (2010) The dual role of the extracellular matrix in synaptic plasticity and homeostasis. Nat. Rev. Neurosci. 11, 735–746 20944663 10.1038/nrn2898

[23] Brigidi G. S., and Bamji S. X. (2011) Cadherin-catenin adhesion complexes at the synapse. Curr. Opin. Neurobiol. 21, 208–214 21255999 10.1016/j.conb.2010.12.004

[24] Cowell J. K. (2014) LGI1: from zebrafish to human epilepsy. Prog. Brain Res. 213, 159–179 25194489 10.1016/B978-0-444-63326-2.00009-0

[25] Mattingly R. R., and Macara I. G. (1996) Phosphorylation-dependent activation of the Ras-GRF/CDC25Mm exchange factor by muscarinic receptors and G-protein β γ subunits. Nature 382, 268–272 8717044 10.1038/382268a0

[26] Corradi A., Fadda M., Piton A., Patry L., Marte A., Rossi P., Cadieux-Dion M., Gauthier J., Lapointe L., Mottron L., Valtorta F., Rouleau G. A., Fassio A., Benfenati F., Cossette P. (2014) SYN2 is an autism predisposing gene: loss-of-function mutations alter synaptic vesicle cycling and axon outgrowth. Hum. Mol. Genet. 23, 90–103 23956174 10.1093/hmg/ddt401 PMC3857945

[27] Onofri F., Giovedi S., Kao H. T., Valtorta F., Bongiorno Borbone L., De Camilli P., Greengard P., Benfenati F. (2000) Specificity of the binding of synapsin I to Src homology 3 domains. J. Biol. Chem. 275, 29857–29867 10899172 10.1074/jbc.M006018200

[28] Weston S., Czieso S., White I. J., Smith S. E., Kellam P., and Marsh M. (2014) A membrane topology model for human interferon inducible transmembrane protein 1. PLoS ONE 9, e104341 25105503 10.1371/journal.pone.0104341 PMC4126714

[29] Tokuyasu K. (1973) A technique for ultracryotomy of cell suspensions and tissues. J. Cell Biol. 57, 551–565 4121290 10.1083/jcb.57.2.551 PMC2108989

[30] Slot J. W., and Geuze H. J. (2007) Cryosectioning and immunolabeling. Nat. Protoc. 2, 2480–2491 17947990 10.1038/nprot.2007.365

[31] McPherson P. S., Takei K., Schmid S. L., and De Camilli P. (1994) p145, a major Grb2-binding protein in brain, is co-localized with dynamin in nerve terminals where it undergoes activity-dependent dephosphorylation. J. Biol. Chem. 269, 30132–30139 7982917

[32] Onofri F., Giovedì S., Vaccaro P., Czernik A. J., Valtorta F., De Camilli P., Greengard P., and Benfenati F. (1997) Synapsin I interacts with c-Src and stimulates its tyrosine kinase activity. Proc. Natl. Acad. Sci. U.S.A. 94, 12168–12173 9342381 10.1073/pnas.94.22.12168 PMC23739

[33] Huttner W. B., Schiebler W., Greengard P., De Camilli P. (1983) Synapsin I (protein I), a nerve terminal-specific phosphoprotein: III: its association with synaptic vesicles studied in a highly purified synaptic vesicle preparation. J. Cell Biol. 96, 1374–1388 6404912 10.1083/jcb.96.5.1374 PMC2112660

[34] Chivian D., Kim D. E., Malmström L., Bradley P., Robertson T., Murphy P., Strauss C. E., Bonneau R., Rohl C. A., and Baker D. (2003), Automated prediction of CASP-5 structures using the Robetta server. Proteins 53, 524–533 14579342 10.1002/prot.10529

[35] Leaver-Fay A., Tyka M., Lewis S. M., Lange O. F., Thompson J., Jacak R., Kaufman K., Renfrew P. D., Smith C. A., Sheffler W., Davis I. W., Cooper S, Treuille A., Mandell D. J., Richter F., Ban Y. E., Fleishman S. J., Corn J. E., Kim D. E., Lyskov S., Berrondo M., Mentzer S., Popović Z., Havranek J. J., Karanicolas J., Das R., Meiler J., Kortemme T., Gray J. J., Kuhlman B., Baker D., Bradley P. (2011) ROSETTA3: an object-oriented software suite for the simulation and design of macromolecules. Methods Enzymol. 487, 545–574 21187238 10.1016/B978-0-12-381270-4.00019-6 PMC4083816

[36] Benkert P., Tosatto S. C., and Schomburg D. (2008), QMEAN: a comprehensive scoring function for model quality assessment. Proteins 71, 261–277 17932912 10.1002/prot.21715

[37] Phillips J. C., Braun R., Wang W., Gumbart J., Tajkhorshid E., Villa E., Chipot C., Skeel R. D., Kalé L., and Schulten K. (2005) Scalable molecular dynamics with NAMD. J. Comput. Chem. 26, 1781–1802 16222654 10.1002/jcc.20289 PMC2486339

[38] Brooks B. R., Brooks C. L. 3rd, Mackerell A. D. Jr., Nilsson L., Petrella R. J., Roux B., Won Y., Archontis G., Bartels C., Boresch S., Caflisch A., Caves L., Cui Q., Dinner A. R., Feig M., Fischer S., Gao J., Hodoscek M., Im W., Kuczera K., Lazaridis T., Ma J., Ovchinnikov V., Paci E., Pastor R. W., Post C. B., Pu J. Z., Schaefer M., Tidor B., Venable R. M., Woodcock H. L., Wu X., Yang W., York D. M., and Karplus M. (2009) CHARMM: the biomolecular simulation program. J. Comput. Chem. 30, 1545–1614 19444816 10.1002/jcc.21287 PMC2810661

[39] Darden T., York D., and Pedersen L. (1993) Particle mesh Ewald: an N-log(N) method for Ewald sums in large systems. J. Chem. Phys. 98, 10089–10092

[40] Ryckaert J. P., Ciccotti G., and Berendsen H. J. C. (1977) Numerical integration of the Cartesian equations of motion of a system with constraints: molecular dynamics of *n*-alkanes. J. Comput. Phys. 23, 327–341

[41] Feller S. E., Zhang Y., Pastor R. W., and Brooks B. R. (1995) Constant pressure molecular dynamics simulation: the Langevin piston method. J. Chem. Phys. 103, 11.

[42] Jacob J., Duclohier H., and Cafiso D. S. (1999) The role of proline and glycine in determining the backbone flexibility of a channel-forming peptide. Biophys. J. 76, 1367–1376 10049319 10.1016/S0006-3495(99)77298-X PMC1300115

[43] Bailey C. C., Kondur H. R., Huang I. C., Farzan M. (2013) Interferon-induced transmembrane protein 3 is a type II transmembrane protein. J. Biol. Chem. 288, 32184–32193 24067232 10.1074/jbc.M113.514356 PMC3820858

[44] Saheki Y., and De Camilli P. (2012) Synaptic vesicle endocytosis. Cold Spring Harb. Perspect. Biol. 4, a005645 22763746 10.1101/cshperspect.a005645 PMC3428771

